# Integrating multi-national teams: over a decade of lessons learned in Chiapas with Partners in Health-Mexico

**DOI:** 10.3389/fpubh.2023.1251626

**Published:** 2024-01-11

**Authors:** Daniel Palazuelos, Hugo Flores, Valeria Macias

**Affiliations:** ^1^Department of Global Health and Social Medicine, Harvard Medical School, Boston, MA, United States; ^2^Division of Global Health Equity, Brigham and Women’s Hospital, Boston, MA, United States; ^3^Partners in Health, Ángel Albino Corzo, Chiapas, Mexico

**Keywords:** community health, global health equity, transformative education, rural health care, anti-colonial action, anti-racist action, public private partnerships

## Abstract

In a globalized world where pathology and risk can flow freely across borders, the discipline of global health equity has proposed to meet this challenge with an equal exchange of solutions, and people working toward those solutions. Considering the history of colonialism, ongoing economic exploitation, and gaping inequities across and within countries, these efforts must be taken with care. The Partners In Health program in Chiapas, Mexico was founded in 2011 by a team of leaders from both the United States and Mexico to strengthen the public health and care delivery systems serving impoverished rural populations. Key to the strategy has been to marshal funding, knowledge, and expertise from elite institutions in both the United States and Mexico for the benefit of an area that previously had rarely seen such inputs, but always in close partnership with local leaders and community processes. With now over a decade of experience, several key lessons have emerged in both what was done well and what continues to present ongoing challenges. Top successes include: effective recruitment and retention strategies for attracting talented Mexican clinicians to perform their social service year in previously unappealing rural placements; using effective fund-raising strategies from multinational sources to ensure the health care delivered can be exemplary; and effectively integrating volunteer clinicians from high-income contexts in a way that benefits the local staff, the foreign visitors, and their home institutions. A few chief ongoing challenges remain: how to work with local communities to receive foreign visitors; how to hire, develop, and appropriately pay a diverse workforce that comes with differing expectations for their professional development; and how to embed research in non-extractive ways. Our community case study suggests that multinational global health teams can be successful if they share the goal of achieving mutual benefit through an equity lens, and are able to apply creativity and humility to form deep partnerships.

## Introduction

“The most radical thing we can do is connect people to one another.” – Rosabeth Moss Kanter

The promise of global health has been that it can inspire and coordinate efforts that will improve an equitable access to health for all. In contrast to colonial medicine, tropical medicine and international medicine, the new and better approach promised by global health has been to confront the systems that allowed for great disparities in health outcomes across the globe, initially most prominently in access to treatment for HIV, but increasingly in access to what has been termed universal health care (1). Nevertheless, these processes inherited centuries of historic injustices and ongoing economic extraction between different groups. These differences have sometimes been envisioned as a Global North and a Global South, with the colonial legacy perpetuated even within countries, such as in the rural–urban divide, or within groups, such as the divides of gender, ability, race, class, ethnicity, LGBTQ+ status, etc. (2) Any group hoping to work thoughtfully with another group that has a different worldview, and that may have been historically marginalized, should understand both the history leading up to that moment and the current position that this history has put each group (3).

This was our understanding when founding Compañeros En Salud (CES), the Mexico branch of the United States non-governmental organization Partners In Health (PIH), currently operating in Chiapas, one of Mexico’s most impoverished and marginalized states. Formally launched in 2011 as a healthcare strengthening organization for rural primary care in partnership with the Mexican government, it drew upon decades of local experience, relationships in the area, and partnerships with institutions far away from Southern Mexico. Most importantly, this included fundraising in the United States and collaborations with universities, either through formal partnerships or more informal coordination, such as Harvard Medical School, the Tec de Monterrey, Universidad Nacional Autonoma de Mexico, la Facultad de Enfermeria y Obstetricia, Notre Dame, and others. This all afforded CES access to increased funding not available to most other groups in the region, and a pool of talented staff that traditionally had not chosen to work in the area. With this foundation of improved inputs, CES was able to more fully implement core components of a functioning health system, what PIH has formulated as “the 5 Ss” (Staff, Stuff, Spaces, Systems, Social Supports) (4).

In addition to offering an improved clinical space for Mexicans, CES has also found a way to include trainees from the United States, and to a lesser extent Europe, looking to incorporate global health rotations into their training. Also known as the short-term experience in global health (STEGH), these rotations grew rapidly in popularity in the first decade of the 21st century, reaching a peak in 2010 when 30.8% of all graduating medical students in the United States reported such experiences (5). Their experiences with STEGHs, however, had been mixed, with many describing challenges arising from medical students being asked to perform outside of their level of training and being traumatized in the process (6), or local hosts feeling that the medical visitors were ill prepared to contribute meaningfully in a context they did not yet understand (7). CES created a system that above all else aimed to respond to the call to “design new instructional and institutional strategies” that can provide transformative educational experiences to health professionals (8). The goal was to design an educational program with clearly delineated roles and responsibilities so that that the experience could be equally as impactful for both Mexican and United States-trainees, and patients. What follows is a detailed account of our experience in building and running the educational aspects of this program, recognizing that what we present is framed by our unique vantage points and biases as co-founders and program leadership.

## Context

Chiapas, is the southernmost state of Mexico. It is blessed with abundant natural wealth, but it also contains some of the most impoverished and marginalized communities in the country. This is not by accident; Chiapas has long been positioned as an agricultural and mining region within Mexico, where industrialization and development have fallen far behind compared to the rest of the country (9). More so, the communities where CES started operations are about 8 hours away from the closest urban centers. Getting there involves traveling for hours on unpaved roads, on the back of pickup trucks that run perhaps once a day, or through unsafe mountain roads where deadly accidents are still all too common (10).

The design of the Mexican Health System intended to cover the entire population through a vast network of primary health centers, present in most communities in the country, with referral capacity to higher level hospitals. According to WHO data, Mexico has a physician density of 24.1 per 10,000 people, compared to 35.5 in the United States (11). However, in neither country doctors are equally distributed. The 6 million people living in Chiapas have access to only two tertiary care level hospitals, and most services are concentrated in the four biggest cities. In hundreds of small communities across the state, care is provided through the smaller health centers, although the most remote places struggle to find permanent medical staff and are sometimes barely functional as a result.

The Mexican ministry of health (MoH) offers contracts available for doctors to work in some of these communities, and promises a higher salary as an incentive, however, it is very rare that doctors decide to work in such communities. A similar phenomenon occurs in rural areas all over the world, including developed countries like the United States (12). In 1935, the Mexican government attempted to address this issue by instituting a year of mandatory social service, where newly graduated doctors are obligated to work for the MoH in order to obtain their medical license (13). The majority of these newly graduated doctors, called *pasantes*, are still placed to this day in rural areas to cover for hired doctor shortages. For most of this program’s history across Mexico, the *pasantes* were the sole providers for one or more communities, usually while having limited access to tools and supervision (14). CES operated by engaging with physicians about to start their social service year and enticing them to work in these communities by offering a package of economic and academic incentives (15). These young physicians were scheduled to graduate from the program each year, but CES has been able to attract new hires to replace graduates and therefore guarantees continuity of care. Many graduates have even opted to stay longer to work with CES in a leadership position or as a clinical supervisor. In general, *pasantes* have limited clinical experience, and although the Mexican health system allows them to oversee their health centers, CES realized early on that they would benefit from support and mentorship from more experienced clinicians. The majority of this clinical supervision is provided continuously by the cadre of clinical supervisors, the majority of whom are recent Mexican graduates of the CES *pasante* program. To augment this foundation of in-house supervision and mentorship, CES also implemented a program for hosting foreign clinicians who could come to participate in a purely teaching capacity.

Since the first class of *pasantes* joined the program in 2013 up to the writing of this paper there have been a total of nearly 100 *pasantes* working in 9–10 primary care clinics, and later participating in a local referral hospital and a maternal waiting home, though staffing for those clinical entities involves even more clinicians. During this time, around 150 foreign medical volunteers have rotated through the clinics. This is within the context of CES hosting over 300 total volunteers, many for longer placements such as recent college graduates and gap year medical students who worked from 6 to 12+ months as research assistants, program coordinators, or office volunteers. See Table 1. The CES program is experienced by participants as a squarely Mexican run clinical program that is enriched by a large cast of rotating foreign visitors whose work is interwoven with daily operations.

**Table 1:** Visitor log: total number of visitors by category since CES formally launched.

Category	Number
HEAL rotating fellow	8
General visitor	20
Clinical volunteer	151
Research volunteers	18
Non-clinical volunteer	121
Uncharacterized	10

## Details about key programmatic elements

Most countries in the world require that clinicians have a domestic license to practice medicine, and Mexico is no exception. Global health projects from the Global North have often been rightfully criticized for sending unlicensed or partially trained staff to treat patients in the Global South (16). Even for fully licensed clinicians, the question remains of whether practitioners for the Global North should be allowed to practice freely in the Global South for only short visits, as the opposite would never be possible (17).

As soon as CES started clinical operations in 2012, it began attracting physicians from the United States who wanted to do global health rotations during their residency. They visit usually for 1 month and are paired with the Mexican doctors at their community clinics. The United States residents act as bedside clinical educators and coaches, working in the spirit of what PIH calls “accompaniment.” This is a philosophical stance, but also a rubric for programmatic design (18). At CES, the Mexican doctors remain responsible for the patients’ care and have the final say about the diagnosis and treatment. Legally, United States residents are acting as visiting students, in the same way that foreign students rotate in the United States, acting always under the supervision of local professionals and therefore contribute without violating any Mexican regulations.

For global health programs in Europe and the United States, it is important to find field placements that fulfill the student’s learning and exposure expectations. The United States residents in CES are mentored by the CES medical director, who serves as a bridge between the practice of medicine in the United States and in rural Mexico. This position has historically been held by both US and Mexican physicians, showing how success in this role is determined not by nationality alone but by the ability to be an inclusive mentor. See Box 1 for further information on how CES structures the visit in order to set clear expectations and facilitate a beneficial experience for the pasante, the United States visitors, and patients. Additionally, even though many of the visiting residents have had only limited experience in resource constrained settings, they are instructed during their orientation on the relative lack of diagnostic and treatment resources in the rural CES clinics. Accommodating to this reality can be a struggle for some, so the CES cadre of clinical supervisors will often pay extra attention to supporting those clinicians when they visit them in the community; through ongoing on-site reflection and discussion, many of these foreign clinicians are able to learn how to provide high-quality clinical care in rural Mexico through the tutelage of their more experienced Mexican colleagues.

**BOX 1 How CES operationalized equitable collaborations in medical contexts.**

United States medical trainees and professionals who were interested in working with CES were given a set of very clear rules of engagement that both clarified the ways they could get involved, and the things they should not do.

Getting there

Each applicant is interviewed in Spanish to see if their values, personal approach, and Spanish-language skills align with CES. Translators are not used, so each participant must have at least a functional level of Spanish. The time that participants contribute is commensurate with their level of expertise: college and medical students are asked to give at least 6–12 months; residents are asked to give at least 4 weeks, and attendings are allowed to visit for less time, usually around 2 weeks. Visitors are not charged for the experience; they only have to pay for their own flight, and upon arriving are picked up and brought to the CES offices by CES staff. There they are given an orientation to the team and the CES approach. It is made very clear that their presence is governed by an overriding and non-negotiable rule: if anyone in the CES staff feels that their presence is not beneficial for the team, they will be driven back to the capital city where they can spend the rest of their time on their own. This is said in a friendly and supportive way, but it makes it very clear who is in charge. As of the publication of this paper, less than 1–2% of all volunteers have acted in ways that led to their disengagement with working with CES.

After the initial orientation in the central coordination site in the larger town of Angel Albino Corzo (locally known as Jaltenango), the visitor is driven out to a rural community by an experienced driver in a vehicle that can safely make the trip. There, they will be greeted by the Mexican *pasante* who has coordinated where they will stay and what they will eat. This usually means living in a local house and eating with a local family in conditions not much different than what the average patient experiences. They will awaken to the sound of roosters and will spend most nights making house calls and drinking sweet coffee by a wood-burning stove. There is limited electricity and little to no internet.

Working together in the clinic

In the clinic, the visiting doctor is instructed to sit to the side of, and slightly behind, the Mexican doctor. They are to watch the clinical interaction but are invited to ask any clarifying questions while the Mexican doctor is gathering their history and conducting a physical. From there, both leave the room and go to an adjacent pharmacy where they will discuss the case. This presents a natural opportunity for the visiting MD to teach about medicine away from the patient who might interpret such an interaction as one MD being superior to another. The Mexican doctor will usually, in turn, educate the visitor on local contextual factors that might affect the diagnosis and treatment plan. After selecting the treatment from the fully stocked pharmacy, both will go back to the examining room, but only the Mexican doctor should explain to the patient the diagnosis and treatment plan.

Formal course work and community

Each month, all the *pasantes* in each of the rural clinics where CES works gather in the central CES offices to restock their pharmacies and to receive part of a course (locally known as “dias de curso”). In addition to reviewing purely clinical and social medicine topics, the course focuses on how broader socio-economic and political factors affect health, what have been called the “actual causes of poverty;” centering these insights, often from the perspective of those in the Global South most affected by these processes, helps to avoid depoliticizing international experiences such they become only an exercise in self-actualization for the already privileged (45). The visiting MDs are given the opportunity to teach a session if they want, and to engage on a social level with the larger team. Despite the serious content covered, this is usually a joyous and educational event and presents an opportunity to get much needed rest, re-energize, and build community. At the conclusion of their time with the CES team, the visiting doctors usually opt to add a few free days to visit local sites before returning home. Many visitors have reported this experience to be a crowning jewel of their medical training, and that the relationships they form are set to last a lifetime.

For care delivery organizations in the global south, it is important to benefit from visitor’s expertise; many patients in CES-supported communities who presented with more complex diagnoses, for example for neurologic or cardiologic conditions, could get access in their local clinic to a team of clinicians thinking about how best to address their health concerns. Otherwise, traveling to see a specialist in the few referral hospitals could be quite expensive, difficult logistically, and may not even result in a favorable outcome. Through this partnership, thousands of patients were able to have access to better care and follow up, without leaving their communities and without losing continuity with their local doctor, which can result in both clinical and economic benefits for the patients. Notwithstanding, any patient who seeks help from CES and ultimately needs specialist care is still accompanied to receive that care, usually in the urban medical centers, through CES’s “Right to Healthcare” program (19).

Table 2 shows select quotes gathered from exit surveys submitted by visiting doctors. These were collected during exit interviews that included opportunities for both verbal and written feedback. Concerns and criticisms were usually discussed and addressed verbally, so written submissions nearly always captured generally positive responses. These do not represent a complete analysis, but rather are included to give a sense of how this program performed at its best. The responses suggest that with proper preparation and mentorship, visiting residents can balance both learning and service without causing harm to patients or engaging in unregulated practice. Exit interview responses from Mexican pasantes were not routinely recorded but in verbal interviews with CES leadership they nearly universally expressed enjoying the company of a fellow physician in their clinics and their communities, having a companion at a time that could otherwise be a terrifying assignment. Both parties regularly expressed feeling that they were supporting and teaching each other. A more complete and rigorous analysis that includes the opinions of all staff, participants and graduates of the program should be conducted. The Brigham and Women’s Hospital IRB reviewed inclusion of the quotes and determined that as presented are not human subjects research (REDcap ID #679).

**Table 2:** Example themes heard from visiting MDs from feedback exit surveys.

Theme	Quote
Mentorship	*“This was an amazing opportunity for me and really separated it from experiences I have had in global health previously… Teaching is what makes the rotation so impactful and the pasantes were open and excited to be taught.”* *“The pasantes and structure of the rotation. I loved working one-on-one with them. I also really enjoyed the dias de curso. I was happy to be able to help with a presentation.”* *“The opportunity to be immersed in the community with the pasante and teach in a responsible way.”*
Clinical experience	*“As for medical knowledge, in the face of a remote rural setting with limited resources, I was exposed to the TRUE ART OF MEDICINE. The resident experience that CES provides is unparalleled and unavailable anywhere in the United States.”* *“[I enjoyed] The house calls in the community-you never knew what you were walking into”*
Community	*“I truly enjoyed my experience in [this community]. I felt well integrated into the community by eating meals with local families and taking part in house visits.”* *“The meals provided [by the host family] were also extremely wonderful and a great way to get to know the people in the community more intimately.”*
Mentorship + Clinic Personnel	*“[The pasante] was absolutely fantastic to work with and included me in medical decision-making. The clinic was well organized by [the pasante and the nurse].”* *“[The community] was an amazingly beautiful community and [the pasante] quickly became a close friend. The nurse was extremely helpful in the clinic and we had everything we needed most of the time. Wonderful experience!”*
Health system	*“I learned so much about how to navigate a health system with few resources. It may have been helpful to be debriefed on PIH practices-pasantes/ acompañantes and the emergency system before going out into the community. I found this to be really interesting once I learned about it as well as integral to our work.”*
CES	*“I enjoyed the people and the environment. There’s a strong sense of community and shared mission that was really contagious.”* *“Getting to know all of the CES “banda” was definitely the best part! To meet everyone, from drivers to supervisors and all in between, was such a great opportunity for networking.”* *“Two things [were my favorite parts]: 1) Getting to spend time with just about everyone involved in CES, mostly due to the course which was also wonderful to experience and participate in and 2) In-community resident experience allowing time for clinical teaching while simultaneously learning so much about the health system.”* *“Working with other people that are so passionate about serving the communities of Chiapas! I appreciated that everyone was open, really knew each other, and really worked together on many different projects.”* *“It is a great community of dedicated people. I enjoyed learning from everyone and getting ideas about how to improve health care, not just in Chiapas but other areas as well.”* *“I believe you guys are doing a great job/service not only to the members of the communities in Chiapas, but also around Mexico by helping to further train the medical students (pasantes). Having them staff patients with you guys and having supervisors is a great idea because you are also contributing to form better physicians, who can then hopefully provide a better quality of care no matter where life takes them to serve later on. I admire you are work and learned very much from the health model you apply. Thank you very much about this experience.”*

### How does CES work to keep local needs central to how it makes decisions and operates?

**Figure 1:**
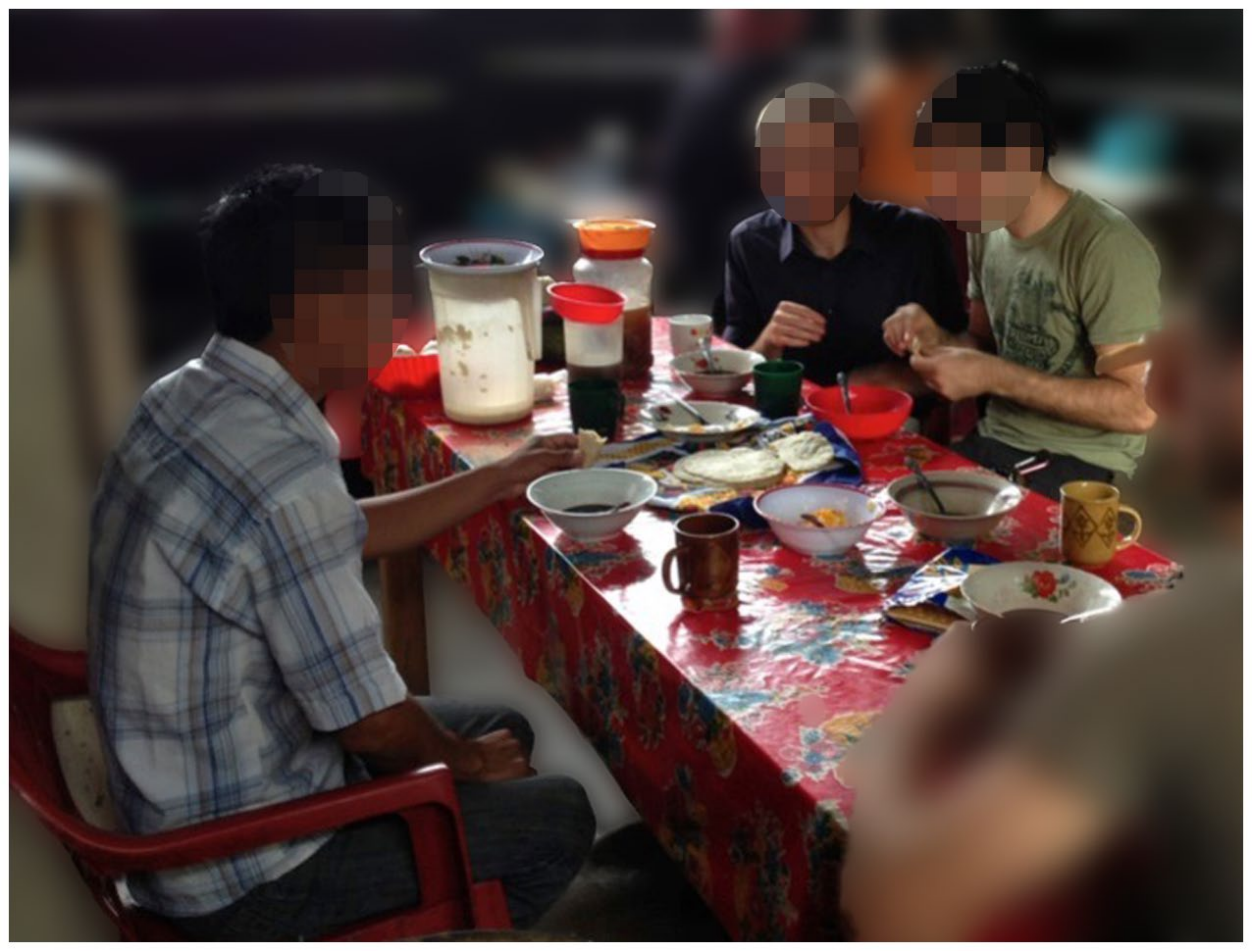
The CES model of training in action. A medical doctor visiting CES from the United States converses with a Mexican doctor completing his social service year and a community health worker active in the area, as they share breakfast in a neighbor’s house prior to a day of work in the community clinic. This meal is prepared by locals, almost always women, but they are appropriately reimbursed and this system has become an important source of revenue for hosts. The families will also often sit and share the meal with the doctor and team, thereby offering an opportunity to share and learn together in an informal domestic setting.

If the leadership, funding, staff and visiting supports are mostly not from the same area as the patients, there is a very real risk that the interventions can be positioned as benevolence or charity, instead of a mutual partnership working together to overcome locally identified problems. CES was designed from the beginning to address such concerns by embedding key programmatic mechanisms in its structure and operations. First, because CES was founded after years of preliminary work in the area, we heard a clear mandate from every individual patient and community leader, be it through local churches, coffee farmer cooperatives or *ejido* groups, that revitalizing defunct government clinics was a priority. In operating the clinics, the *pasantes* were expected to live in the communities for most of the month, instead of leaving every weekend to hand in paperwork to the jurisdiction offices; this required forming an agreement with the jurisdiction leaders to accept such a rhythm of data flow, but it also allowed the *pasantes* working with CES to fully immerse themselves in local life. As such, they were also shown how to rent a room and eat their meals in local houses, always paying a just wage to the hosting families for that support (See Figure 1). Similarly, when launching a new program, such as a community health program (20) or a shared medical appointment program for diabetes (21), the *pasantes* were encouraged to formulate their plans with local leaders, such as elected officials and community health committees.

CES leadership made an early tactical decision to not put the head offices in the state capital or the more comfortable tourist towns, billed as magical places, or “pueblos magicos.” This was because we knew that proximity would facilitate more and better conversations with the communities we were serving. As such, all of the support staff, from drivers and cooks to accountants and office managers, were hired from the local area, and they have remained some of the most continuously employed members of CES. The organizational culture was purposefully maintained as being very horizontal, with every staff member being encouraged to share any of their ideas and concerns with top leadership at any time. Although it would be impossible to immediately act upon every report, this structure opens the opportunity for frontline insights being available to directly influence strategy and implementation tactics. Finally, CES implemented a NGO-run pilot community health worker program early on, and developed the program to both be a source of support for patients with chronic diseases and a direct pipeline of contact with community members. The structure of this program is further outlined in other publications, and recently has undergone a transformation toward professionalizing the CHWs’ position at CES (22). PIH has long worked under the premise that “community health workers are the biggest defense against the ignorance of the expert” (23) but this is only if what they say is heard. Many of the CHWs, nearly all of them women, grew in their leadership roles in the communities because of their work with CES, and as such have been increasingly more valuable allies to CES. The ultimate goal is that the experiences with this program influence the broader national conversations on community health in Mexico.

Similarly, while the visiting residents are valued, they are not positioned as indispensable. Of note, the addition of visiting foreign residents did not erode or make the CES system dependent on them, as the quantity of patients seen by the pasante-resident team doctors was the same as when the pasante was alone. Similarly, after observing that resident collaborators function best when they can engage in long-term relationships through repeat rotations, CES has worked to facilitate agreements with United States training programs so that the best residents can rotate more than once.

### What are ongoing challenges that remain difficult to fully address?

#### Questions about the presence of foreigners

As with any intervention, there will always be countervailing forces and unintended consequences. In the communities, there was an initial period of adjustment with so many foreign faces suddenly showing up to a community that had previously almost never seen foreigners. Similarly, despite all the efforts taken to keep the Mexican doctor central to care delivery, some patients still request that they be seen by the foreign doctor in the mistaken belief that they might get better care. This was interpreted as a key example of internalized bias. These challenges were confronted directly by discussing with community members, either individually or in community fora, the reasons why foreign doctors were visiting. When patients later saw these visiting doctors living in the communities in the spirit of humility and service, this went a long way to win trust and form positive working relationships. For CES, the working principle is that the health system can win trust by working as a health system that aims to be trustworthy.

Of note, the Mexican doctors who work and train with CES often come from the middle-and upper-middle class of Northern Mexico, and are often taller and lighter in skin tone when compared to the Chiapanecan patients. As such they are sometimes lumped together with the other “gringos” (a complex term that can be spoken as a disparaging term or used simply as an identifier, but we include it here because it is often used by community members). Most of the Mexican doctors from Northern states have been surprised to hear this. When they protested, some local patients have humorously applied the term “gringos con pelo negro” (gringos with black hair) for the Mexican doctors. On the other hand, some of the doctors from Chiapas, who often look and speak more like the local population, have expressed that they will sometimes have to work to come across as equally as credible as the newly arrived doctors. In a confrontation between region, race and class, the idealistic Mexican doctors who came to work with CES learn important lessons about the axes of difference that can exist even within a national territory.

A related concern is how to facilitate diversity in the visiting professionals. For the doctors, this includes diversity in race, class, and region of the United States; since global health does not have a federal funding mandate in the United States, it remains mostly, but not exclusively, prevalent in elite private coastal institutions. Similarly, beyond United States trainees, CES has benefited tremendously from the perspectives and experiences offered by visitors from other countries and continents, such as Haiti and other parts of Latin America, Asia or Africa. Current funding availability makes such visitors far less common. Beyond doctors, CES recognizes that a similar program with other cadres of health workers, such as nurses and advanced practice practitioners (APPs), would bring great value; despite an initial (24) and sustained focus on nurse training in CES, United States nurses and APPs have far fewer opportunities to participate in global health rotations abroad.

#### Facilitating long-term career options for CES graduates

Many of the Mexican and visiting doctors have expressed interest in working with CES as a career option long-term. In fact, the training program has served as a powerful recruitment tool for CES as it hires new cadres of supervisors and program directors. We interpret this as a success because CES has been able to inspire doctors to continue working in a rural area that has normally faced severe staffing shortages. Setting equitable salaries for these full-time professional staff continues to be an ongoing negotiation, especially if a United States-trained and Mexico-trained doctor are hoping to do similar work but have different expectations about their salaries. In short, United States-trained physicians can make over 10x the salary of a Mexican if they stay in the United States. Many need to make this amount if they hope to pay for their student loans and save enough to retire in the United States (25); the average medical debt for United States medical graduates in 2022 was $205,307 USD (26). At the same time, it would be deeply unfair for the doctors who train in Mexico to make dramatically less when compared to their United States-trained peers simply because of their country of origin. Similarly, the most talented Mexican graduates usually have options to make a salary similar to that seen in the United States if they work in the private sector in Mexico, thereby putting them in a similar situation to their United States-trained counterparts if they choose to work with CES in rural Chiapas. As such, there must be a constant negotiation between how much different cadres of doctors can be expected to lower or increase their salary expectations (27, 28). Some organizations have addressed this challenging situation by creating single salary scales and adjusting salaries according to the cost of living, instead of the nationality of staff. Global health organizations have the potential to become great equalizers when it comes to stop perpetuating the labor practices that facilitate staff from the Global North and in the private sector to develop fruitful careers, while workers from the Global South and in the public sector remain positioned as laborers. Solving these inequities will require both thoughtful management solutions within international organizations, and an increase in the total funding available to such organizations.

### How to best embed research into the care delivery program

Another core area of tension remains the value of research. It has been well described that too much research is not actually returning enough benefit to source contexts, and is often not available when locked behind paywalls (29), and in the era of Open-Access publishing may favor well-funded authors disproportionately having their voices heard over authors who struggle to pay the publication fees. As such, there is a call for the practice of extractive global health research to end, such that a more inclusive and collaborative research practice can take its place (30). This ideal would be marked by published works that reflect the priorities and narratives of those who stand to be most affected by the impact of the research, such as through policy change inspired by the findings. There are important systemic factors that make reaching this ideal difficult; for many United States-based researchers, if they do not publish, they will not be promoted in their academic institutions, or they may lose their jobs altogether. This creates a false urgency in extracting data and processing it for publication. Working in impoverished contexts to build up local capacity to control their own research and advocacy agenda takes time. Few to no United States academic medical centers currently put value on this process of building local capacity as a criterion for promotion (31). At CES, despite early examples of rigorous implementation science being embedded into care delivery, such as an early version of the CHW program being rolled out as a stepped-wedge trial (32, 33), service and care expansion ultimately took precedence over research. Producing scholarship, however, has remained an important priority, and has been driven by the doctors and collaborators working most closely with the communities to build their own research agenda, especially after gaining further training in research methods, such as through participation in the HEAL initiative (34) or through getting further education in the United States or Europe and then returning to equity-focused work (35), either in Chiapas or similar contexts. Ironically, even if Mexican researchers can control the research agenda, they will find that they must still choose between publishing in their own language or aim to publish in English with the goal that this will lead to greater influence.

## Discussion

CES was launched from a mixture of early experiences in the area (36), new energy sparked by the rise of global health as a discipline (37), and the need to meet increasing student demand for meaningful experiences in the field (38). Invigorated by the moral clarity that health is a human right, we as co-founders and leadership of CES recognized that by marshaling the necessary resources, we could build a health care delivery and educational system that would be locally transformative and internationally relevant. Now with over a decade in operation, CES continues to grow. This has included expansion into a maternal waiting home to provide more person-centered birthing care, revitalization of one of the referral hospitals, and during the COVID-19 pandemic, a renewed focus on provider safety and access to intensive care (39, 40). The CES experience suggests that privilege can be leveraged to redistribute capacity and human resources, and build systems that extend improved care to communities that need to be supported on a priority basis. If well designed and managed, this enterprise can be used as a beacon experience for training the next generation of professionals who hope to do similar work. The convening force of such a program can also generate a common and shared space that builds a multi-national community of change agents who can continually turn to each other for advice and inspiration.

It is important, however, for any organization to reflect at regular intervals, such as after the collective trauma that was the COVID-19 pandemic, to reconsider why and how the work is being pursued. The most recent 2023 AAMC graduation survey discovered that United States-based medical students now self-report a level of participation in global health experiences that is nearing a low rate not seen in nearly three decades; after a height at 30.8% in 2010, the rate dropped slightly in 2019 to 24.2%, but then continued dropping to 14.2% in 2022, and finally reached a new low of 11.3% in 2023 (41). The reasons for this are likely many, but we suspect that many United States and European students have recognized that they can pursue their professional interests in equity by working domestically, especially considering the important and active struggles that arose from the murder of George Floyd, the repeal of reproductive rights in the United States, and the rise of far-right extremism that increasingly pushes anti-immigrant rhetoric and policy, etc. Many others are concerned about the environmental impact of international flight to participate in only a short-term experience (42). There is also an ongoing conversation on how to best embed anti-racism and anti-colonialism praxis in global health (43).

The CES experience shows how a clinical and educational program can be built to structure equitable interactions between people who may be dissimilar in terms of nationality, region, race, class, gender, ability, and other axes of difference. The tactical design principles described here can serve others hoping to launch similar programs. A lot of attention has been paid to the potential unintended consequences of work in global health (44). There still remain, however, many threats to human health globally that are the result of actions by actors who have all the intention to gain profit regardless of the effects on people or planet. The CES experience suggests that global health does not need to abandon finding ways to work together. Instead, global health efforts can be structured to unite like-minded individuals in multi-national teams to work in common cause as we confront the biggest threats to our common future, but always by centering the perspectives of those in the Global South who stand to be most affected by these threats.

## Acknowledgment of any conceptual constraints

This report was written by the co-founders and current leadership of CES to be a descriptive tour of what we see as the key lessons from our experience. We are aware, however, that these descriptions are tainted by our personal investments in the program. While there are many benefits to how we have chosen to work, there are alternative models for pursuing social change, and we invite the discussion comparing and contrasting different approaches.

## Data availability statement

The original contributions presented in the study are included in the article/supplementary material, further inquiries can be directed to the corresponding author.

## Ethics statement

Ethical approval was not required for the study involving human participants in accordance with the local legislation and institutional requirements. As the Mass General Brigham IRB determined that the content and activities presented in the manuscript do not constitute human subject research, written informed consent for participation was not required in accordance with the national legislation and the institutional requirements. Written informed consent was obtained from the photographed individuals for the publication of identifying images.

## Author contributions

DP and HF wrote the original draft. VM provided conceptual and editorial support in writing the manuscript. All authors contributed to the article and approved the submitted version.
